# Detection of *a* and *b* waves in the acceleration photoplethysmogram

**DOI:** 10.1186/1475-925X-13-139

**Published:** 2014-09-25

**Authors:** Mohamed Elgendi, Ian Norton, Matt Brearley, Derek Abbott, Dale Schuurmans

**Affiliations:** Department of Computing Science, University of Alberta, 2-32 Athabasca Hall, T6G 2E1 Edmonton, Canada; National Critical Care and Trauma Response Centre, Darwin, Australia; School of Electrical and Electronic Engineering, University of Adelaide, Adelaide, SA 5005 Australia

**Keywords:** Vascular effects, Hypertension, Atherosclerosis

## Abstract

**Background:**

Analyzing acceleration photoplethysmogram (APG) signals measured after exercise is challenging. In this paper, a novel algorithm that can detect *a* waves and consequently *b* waves under these conditions is proposed. Accurate *a* and *b* wave detection is an important first step for the assessment of arterial stiffness and other cardiovascular parameters.

**Methods:**

Nine algorithms based on fixed thresholding are compared, and a new algorithm is introduced to improve the detection rate using a testing set of heat stressed APG signals containing a total of 1,540 heart beats.

**Results:**

The new *a* detection algorithm demonstrates the highest overall detection accuracy—99.78% sensitivity, 100% positive predictivity—over signals that suffer from 1) non-stationary effects, 2) irregular heartbeats, and 3) low amplitude waves. In addition, the proposed *b* detection algorithm achieved an overall sensitivity of 99.78% and a positive predictivity of 99.95%.

**Conclusions:**

The proposed algorithm presents an advantage for real-time applications by avoiding human intervention in threshold determination.

## Introduction

Although the clinical significance of Accelerated Plethysmograph (APG) measurement has been well-investigated [1–4], there are still a lack of studies focusing on the automatic detection of *a* and *b* waves in APG signals. However, Matsuyama [5] attempted to determine which of the nine QRS algorithms of Friesen’s ECG algorithms [6] suit the detection of a waves in APG signals—this is because the morphology of the R peak in ECG signal is similar to the *a* wave in the APG signal. The detection rate was below 63% for all nine algorithms, even after modifying the thresholds with different values. Matsuyama [5] recommended that a new robust algorithm be developed for both APG and ECG signals. Therefore, our investigation herein is aimed at developing a robust algorithm to detect *a* waves in APG signals and to compare its performance with the prior nine *a* detection algorithms [5]. Up to the present there has been no attempt to detect *b* waves in APG signals; and therefore a new method for detecting the *b* wave is now introduced. To validate the robustness of the developed algorithms, noisy APG signals—measured at rest and after exercise—were tested.

**Figure 1:**
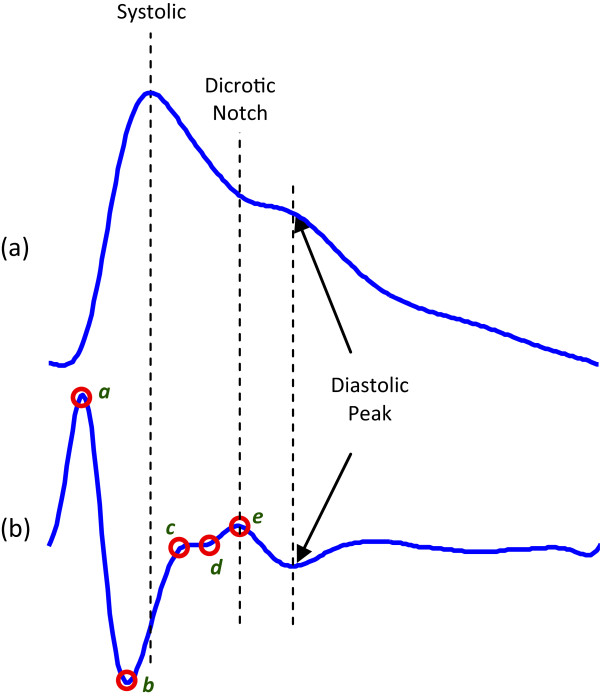
**Fingertip photoplethysmogram signal measurement [**[8]**].** **(a)** Fingertip photoplethysmogram. **(b)** Second derivative wave of photoplethysmogram. The photoplethysmogram waveform consists of one systolic wave and one diastolic wave, while the second derivative photoplethysmogram waveform consists of four systolic waves (*a*, *b*, *c*, and *d* waves) and one diastolic wave (*e* wave).

Photoelectric plethysmography is the most commonly used method for pulse-wave analysis—it is also know as photoplethysmography (PTG/PPG) or digital volume pulse (DVP) analysis. In this paper, the acronym PPG will be used throughout, as recommended by [7]. Fingertip PPG mainly reflects the pulsatile volume changes in the finger arterioles, as shown in Figure 1. Application of the second derivative is typically applied to accentuate subtle changes in the PPG contour [1]. It is the second derivative of the PPG signal that is the APG, also known as SDPPG [7].

As depicted in Figure 1, the APG waveform comprises four systolic waves (*a*, *b*, *c*, and *d* waves) and one diastolic wave (*e* wave) [2]. In our study, the height of detected *a* and *b* waves was measured from the baseline—the values of *a* waves are positive (above the baseline), while the values of *b* waves are negative (under the baseline). The main focus of this study is to provide a robust detection algorithm for *a* and *b* that can be used in clinical studies, e.g., carotid distensibility and ageing [9].

## Materials and methods

### Ethics statement

There is one annotated APG database available at Charles Darwin University. The data were collected during rest (before exercise) and after one hour of exercise (walking) on a treadmill in the climate control chamber at Northern Territory Institution of Sport (Darwin, Australia). The speed of treadmill was set to 5 km/h with a one percent incline increment corresponding to the effort required to walk with 8 kg of webbing. The exercise was considered to be of moderate intensity, and the background of the entire project can be found in [5]. All subjects provided written informed consent before participation, which was approved by the Charles Darwin University Ethics Committee. The database is available upon request at Charles Darwin University: http://www.cdu.edu.au/ehse.

### Data used

The PPGs of 27 healthy volunteers (males) with a mean ± SD age of 27 ± 6.9 were measured using a photoplethysmography device (Salus APG, Japan), with the sensor located at the cuticle of the second digit of the left hand, in which all subjects were included. Measurements were taken while the subject was at rest on a chair. The PPG data were collected at a sampling rate of 200 Hz and the duration of each recording was 20 seconds. The PPG recordings of 20 seconds are intentionally much shorter than is usual for ECG recordings to exclude motion artefacts and other noise [10]. This also serves as a preliminary test of feasibility, where the ease of shorter recording lengths is desirable in a clinical setting.

The annotations were carried out by only one PPG specialist, which is sufficient for this preliminary proof-of-concept study. The signals measured during rest, before exercise, contained a total of 584 heartbeats, whilst the PPG signals collected after one hour of exercise with a total of 885 heartbeats, and two hours of exercise with total of 956 heart beats; the background of the entire project can be found in [5]. For signal analysis and wave detection, MATLAB 2010b (The MathWorks, Inc., Natick, MA, USA) was used.

#### Training set

The PPG signals collected after 1 hour of exercise were used for training as they includes different shapes of PPG waveforms and noise. Moreover, it contained fast rhythm PPG signals, with a total of 885 heart beats, which had an impact on the detection accuracy.

#### Test set

PPG signals were measured at rest (before the exercise), with a total of 584 heart beats, and after 2 hours of exercise, with a total of 956 heart beats, were used for testing.

### Methodology

We discuss and evaluate nine algorithms that are used by Matsuyma [5] to detect *a* waves in APG signals, and introduce a new algorithm that demonstrates greater robustness and accuracy for the *a* wave detection under conditions of heat stress. All of the algorithms we evaluate are advantageous in that they do not impose an extensive computational overhead that is often required in biosignal analysis.

In describing the algorithms for *a* wave detection in this article, note that *X*[ *n*] refers to the raw PPG signal; while *S*[ *n*] refers to the filtered *X*[ *n*] signal. Here, THR_1_, THR_2_, and THR_3_ refer to the the first, second, and third threshold, consequently.

#### Nine algorithms

1. **AF1**: This algorithm is based on the algorithm developed by Morizet-Mahoudeaux [11]. The algorithm examines the amplitudes and slopes of the APG signals, which form the distinctive feature of the *a* wave, exceed certain thresholds. The slope is the first derivative of the APG signal, *S*[ *n*]=*X*[ *n*+1]−*X*[ *n*−1], followed by three fixed thresholds. The optimal values of these thresholds are defined by Matsuyama [5] as follows: THR_1_=0.31 max(*X*[ *n*]), THR_2_=0.0001, and THR_3_=−0.001.
2. **AF2**: This algorithm is based on the algorithm developed by Fraden and Neuman [12]. The algorithm examines the amplitudes and the slopes of the APG signal. The optimal threshold values for the amplitudes and slopes (positive) are THR_1_=0.21 max(*X*[ *n*]) and THR_2_ = 0.75 [5]. The APG signal *X*[ *n*] is rectified. The absolute value of the APG signal is taken as *X*_1_[ *n*]=|*X*[ *n*]|. This signal *X*_1_[ *n*] is then modified using the amplitude threshold THR_1_ as follows: *X*_2_[ *n*]=*X*_1_[ *n*] if *X*_1_[ *n*]>THR_1_ and *X*_2_[ *n*]=THR_1_ if *X*_1_[ *n*]<THR_1_. Then, the first derivative of *X*_2_[ *n*], *S*[ *n*] is calculated as *S*[ *n*]=*X*_2_[ *n*+1]−*X*_2_[ *n*−1], followed by a threshold *S*[ *n*]>THR_2_.
3. **AF3**: This algorithm is based on Gustafson’s algorithm [13]. This algorithm not only examines the positive slopes but also the product of the slope and amplitude of the APG signal. The first derivative *S*[ *n*] is defined as *S*[ *n*]=*X*[ *n*+1]−*X*[ *n*−1], followed by an optimal threshold value THR_1_=62 [5].
4. **FD1**: The concept for this algorithm was taken from Menrad [14]. This algorithm examines the slopes of the APG signal. Menard defined the first derivative as follows: *S*[ *n*]=−2*X*[ *n*−2]−*X*[ *n*−1]+*X*[ *n*+1]+2*X*[ *n*+2], followed by an optimal slope threshold THR_1_=0.099 max(*S*[ *n*]).
5. **FD2**: This algorithm is based on the method developed by Holsinger [15]. The algorithm examines the slopes of the APG signal. The first derivative *S*[ *n*] is described as: *S*[ *n*]=*X*[ *n*+1]−*X*[ *n*−1]. The optimal threshold value for the positive slopes is: THR_1_=150 [5].
6. **FS1**: This algorithm is a simplified version of the technique presented by Balda [16]. The first and second derivatives of the APG signal are employed. The following absolute values of the first and second derivative of the APG signals are obtained by *X*_1_[ *n*]=*X*[ *n* + 1]−*X*[ *n* − 1] and *X*_2_[ *n*]=*X*[ *n* + 2]−2*X*[ *n*]+*X*[ *n* − 2]. The filtered PPG signal is calculated using these derivatives as follows: *S*[ *n*]=1.3*X*_1_[ *n*]+1.1*X*_2_[ *n*], followed by an optimal threshold value THR_1_=154.5 [5].
7. **FS2**: This algorithm adapts the QRS detection technique developed in 1983 by Ahlstrom and Tompkins [17]. This algorithm examines the first and second derivative of the APG signal. The rectified first derivative is calculated as *X*_1_[ *n*]=*abs*(*X*[ *n*+1]−*X*[ *n*−1]). The rectified first derivative is then smoothed as *X*_2_[ *n*]=(*X*_1_[ *n*−1]+2*X*_1_[ *n*]+*X*_1_[ *n*+1])/4. The absolute value of the second derivative is calculated as *X*_3_[ *n*]=*X*[ *n*+2]−2*X*[ *n*]+*X*[ *n*−2]. The smoothed absolute values of the first derivative are added to the absolute values of second derivative as follows *Y*[ *n*]=*X*_2_[ *n*]+*X*_3_[ *n*], followed by two thresholds THR_1_=0.1 max(*S*[ *n*]) and THR_2_=0.8 max(*S*[ *n*]).
8. **DF1**: This algorithm is adapted from the one developed by Engelse and Zeelenberg [18]. The algorithm employs digital filters, such as a differentiator and a low-pass filter. A differentiator is applied to the APG signals *X*_1_[ *n*]=*X*[ *n*]−*X*[ *n*−4], then passed through a digital lowpass filter *S*[ *n*]=*X*_1_[ *n*]+4*X*_1_[ *n*−1]+6*X*_1_[ *n*−2]+4*X*_1_[ *n*−3]+*X*_1_[ *n*−4], followed by a threshold THR_1_=21.
9. **DF2**: This algorithm is based on Okada’s QRS detection algorithm [19]. The algorithm uses digital filters, such as a moving average filter and a low-pass filter. The first step is to smooth the APG signals with a three-point moving average filter *X*_1_[ *n*]=*X*[ *n*−1]+2*X*[ *n*]+*X*[ *n*+1]. Then pass *X*_1_ through a low-pass filter as follows , where *m*=3. The next step is squaring the difference between the input *X*_1_[ *n*] and output *X*_2_[ *n*] of the low-pass filter *X*_3_=(*X*_1_[ *n*]−*X*_2_[ *n*])^2^, followed by a filtering step . A modification step is done as follows *X*_5_[ *n*]=*X*_4_[ *n*], if [ *X*_1_[ *n*]−*X*_1_[ *n*−*m*] ][ *X*_1_[ *n*]−*X*_1_[ *n*+*m*] ]>THR_1_, otherwise *X*_5_[ *n*]=0, where THR_1_=1. The last step is thresholding with THR_2_=0.006 max(*X*_5_[ *n*]) [5].

#### Proposed method

**Figure 2:**
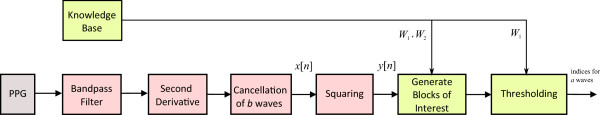
**Flowchart of the knowledge-based** ***a*** **wave detection algorithm.** The algorithm consists of three stages: pre-processing (bandpass filter, cancellation of *b* waves, and squaring), feature extraction (generating blocks of interest based on prior knowledge), and thresholding (based on prior knowledge).

In this study, a novel algorithm, adapted from the framework proposed by Elgendi for detecting systolic waves in PPG signals [20], for detecting QRS complexes in ECG signals [21, 22], and for detecting *c*, *d*, and *e* waves in APG signals [23], will be evaluated. The same approach will be used here to detect the *a* waves. The method consists of three main stages: pre-processing (bandpass filtering and squaring), feature extraction (generating potential blocks using two moving averages), and classification (thresholding). The structure of the algorithm is given in Figure 2.

#### Bandpass filter

**Figure 3:**
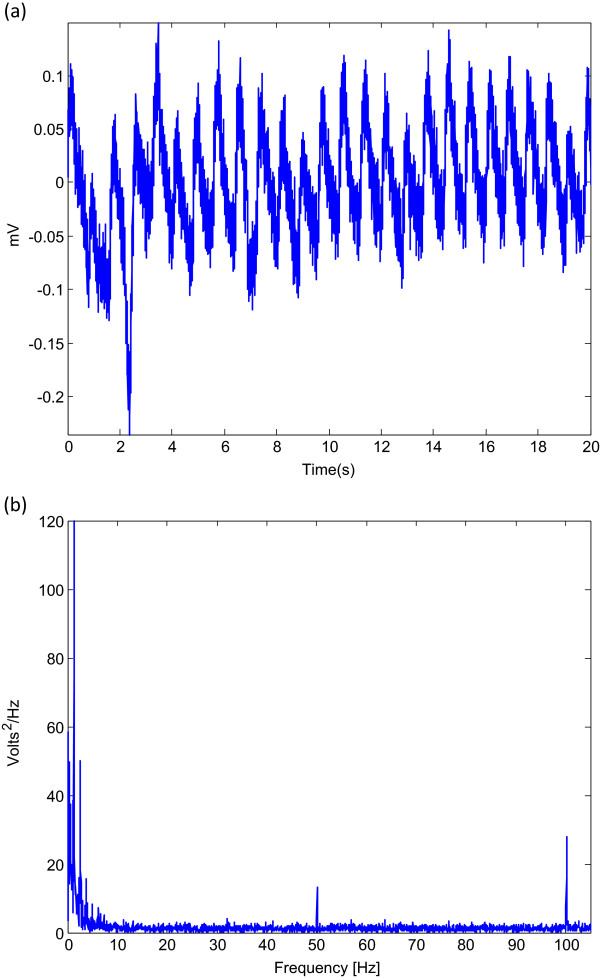
**Fourier transform of noisy PPG signals: (a) PPG signal and (b) Fourier transform (spectrum) of the PPG signal.** The spectrum illustrates peaks at the fundamental frequency of 50 Hz, as well as the second and third harmonics at 100 Hz. The spectrum shows that the main energy of the PPG signal lies below 20 Hz.

**Figure 4:**
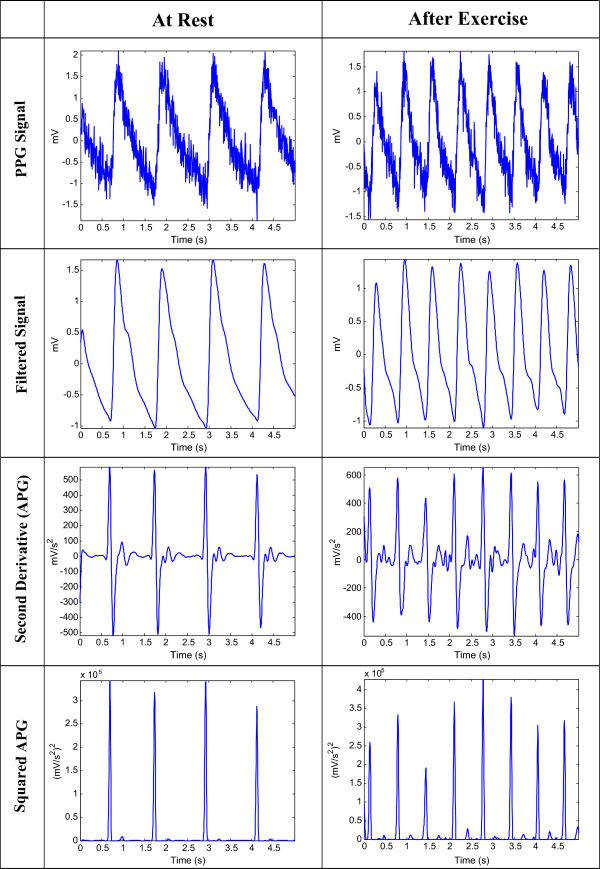
**The proposed algorithm output for PPG measured at rest and after exercise.**

A zero-phase second-order Butterworth filter, with bandpass 0.5–15 Hz based on a brute force search that will be discussed later in the Parameter optimization section, was implemented to remove the baseline wander and high frequencies that do not contribute to the *a* wave (cf. Figure 3). The output of the zero-phase Butterworth filter applied to the PPG signal—at rest and after exercise—produced a filtered signal *S*[ *n*], as shown in Figure 4. The code line of this step is line 2 in the pseudocode of the *a* detection algorithm (Algorithm 1), where *F*_1_=0.5 Hz and *F*_2_=15 Hz.

#### Second derivative

To obtain the APG signals, the second derivative was applied to the filtered PPG in order to analyze the APG signals. Equations 1 and 2 represent a non-causal filter; the three-point centre derivative was created with a delay of only two samples.
12

where *T* is the sampling interval and equals the reciprocal of the sampling frequency and *n* is the number of data points. Figure 4 shows the second derivative of the filtered PPG signal measured at rest and after exercise. The code line of this step is line 3 in the pseudocode of the *a* detection algorithm (Algorithm 1).

#### Cancellation of b wave

At this stage, the *a* wave of the APG needs to be emphasized to distinguish it clearly for detection. This can be done by clipping the negative parts of the APG signal (*Z*[ *n*]=0, if *Z*[ *n*]<0). The code line of this step is line 4 in the pseudocode of the *a* detection algorithm (Algorithm 1).

#### Squaring

Squaring emphasizes the large differences resulting from the *a* wave, which suppress the small differences arising from the diastolic wave and noise, as shown in Figure 4. This step results in the output
3

which is important for improving the accuracy in distinguishing the *a* wave segment in APG signals. The code line of this step is line 5 in the pseudocode of the *a* detection algorithm (Algorithm 1).

#### Generating blocks of interest

Blocks of interest are generated using two event-related moving averages that demarcate the *a* wave and heartbeat areas. The particular method used to generate blocks of interest has been mathematically shown to detect systolic waves [20] and QRS complexes [21].

**Figure 5:**
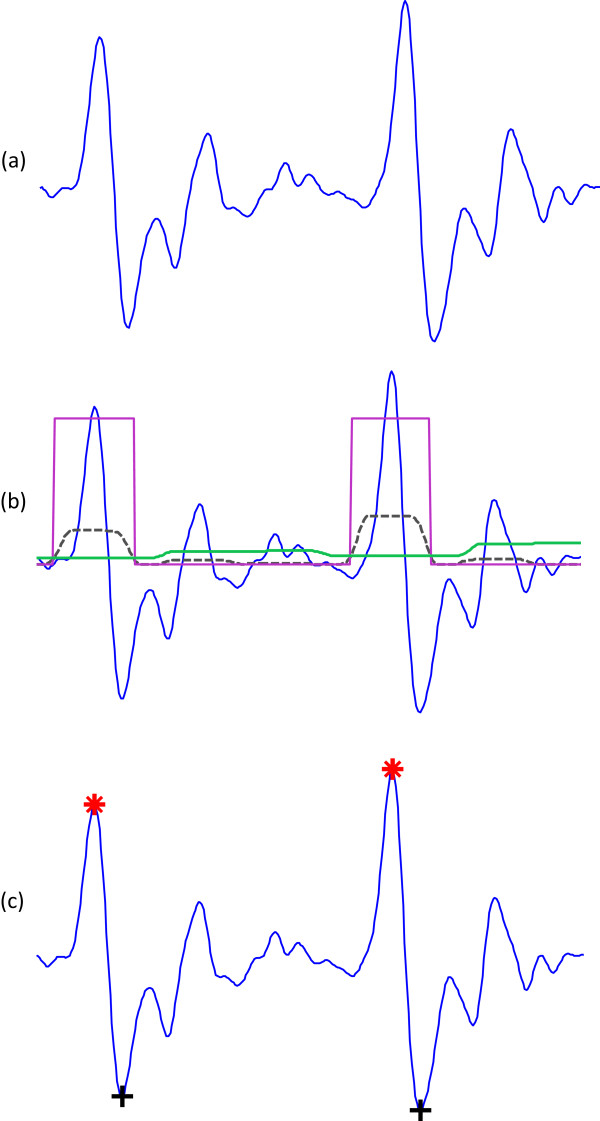
**Demonstrating the effectiveness of using two moving averages to detect** ***a*** **and** ***b*** **waves.** **(a)** Two beats APG signal; **(b)** generating blocks of interest after using two moving averages: the dotted black line is the first moving average MA_peak_ and the solid green line is the second moving average MA_beat_; and **(c)** the detected *a* and *b* waves after applying the thresholds. Here, ‘red asterisk’ represents the detected *a* wave and ‘black plus sign’ represents the detected *b* wave by the proposed algorithm.

In this procedure, the first moving average (MA_peak_) is used to emphasize the *a* wave area, as the dotted signal shows in Figure 5, and is given by
4

where *W*_1_ represents the window size of the systolic-peak duration. The resulting value is rounded to the nearest odd integer. The exact value for *W*_1_ of 175 ms is determined after a brute force search, which will be discussed later in the Parameter optimization section.

The second moving average (MA_beat_) is used to emphasize the beat area to be used as a threshold for the first moving average, shown as a dashed signal in Figure 5, and is given by
5

where *W*_2_ represents a window size of approximately one beat duration. Its value is rounded to the nearest odd integer. The exact value for *W*_2_ of 1000 ms is determined after a brute force search, which will be discussed later in the Parameter optimization section. The code lines of this step are lines 6–7 in the pseudocode of the *a* detection algorithm (Algorithm 1).

#### Thresholding

The equation that determines the offset level (*α*) is , where *β* = 0 based on a brute force search that will be discussed later in the Parameter optimization section, while  is the statistical mean of the squared filtered PPG signal. The first dynamic threshold value was calculated by shifting the MA_beat_ signal with an offset level *α*, as follows:
6

In this stage, the blocks of interest were generated by comparing the MA_peak_ signal with THR_1_, in accordance with the lines 10–17 the code lines shown in the pseudocode of Algorithm 1. Many blocks of interest will be generated, some of which will contain the APG feature (*a* wave), while others will primarily contain noise. Therefore, the next step is to reject blocks that result from noise. Rejection is based on the anticipated systolic-peak width. In this paper, the undesired blocks are rejected using a threshold called THR_2_, which rejects the blocks that contain diastolic wave and noise. By applying the THR_2_ threshold, the accepted blocks will contain *a* waves only,
7

As discussed, the threshold THR_2_ corresponds to the anticipated *a* wave duration. If a block is wider than or equal to THR_2_, it is classified as an *a* wave. If not, it will be classified as noise. The last stage is to find the maximum absolute value within each block to detect the *a* wave; the code lines of this step are lines 19–26 in the pseudocode of the *a* detection algorithm (Algorithm 1). Consecutive *a* waves are shown in Figure 5 to demonstrate the idea of using two moving averages to generate blocks of interest. Not all the blocks contain potential *a* waves; some blocks are caused by noise and need to be eliminated. Blocks that are smaller than the expected width for the *a* wave duration are rejected. The rejected blocks are considered to be noisy blocks and the accepted blocks are considered to contain an *a* wave. The detected *a* waves are compared to the annotated *a* waves to determine whether they were detected correctly. The search range for the true *a* wave was fixed to ± 50 ms for all algorithms to ensure consistency of comparison.

#### Detection of b waves

**Figure 6:**
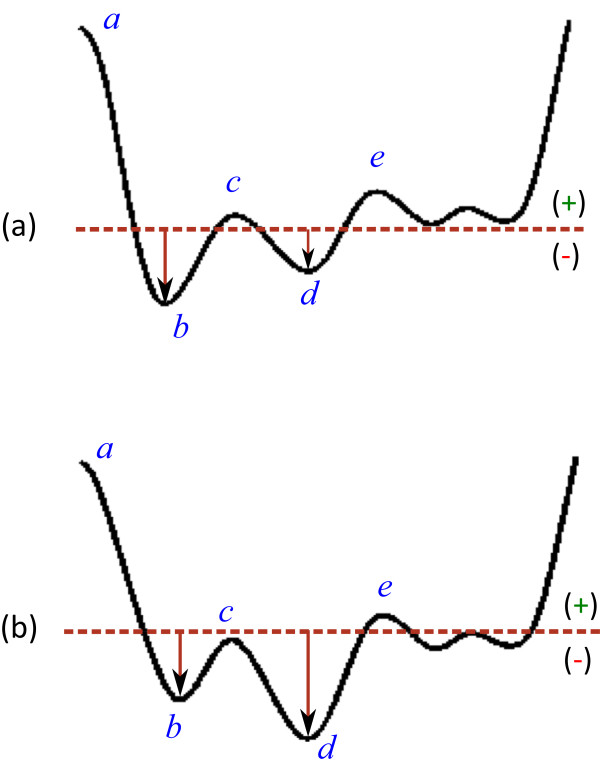
**Demonstrating the local minimum and global minimum of the** ***b*** **wave in the APG signa. (a)** *b* wave is global minimum, **(b)** *b* wave is local minimum.

Figure 6(a) shows the *b* wave as a global minimum in a subject with good circulation, while Figure 6(b) shows the *d* wave as a global minimum in a subject with poor circulation—blood flow becomes restricted to certain parts of the body such as the fingers [24]. However, in both cases, the *b* wave is the first minimum after the *a* wave. The *b* wave can therefore be detected by finding the local minimum, as follows:
8

where APG is the second derivative of the PPG signal (calculated in line 3 in Algorithm 1), *i* is a counter for the detected *a* waves, *k* is the search interval for the *b* waves, and ∧ is logical AND operator. To reduce the computational complexity for finding b waves, the interval *k* has been set to vary from 8 ms to 136 ms.


#### Parameter optimization

Performance of *a* wave detection algorithms is typically evaluated using two statistical measures: sensitivity (SE) and positive predictivity (+P); whereas SE=TP/(TP+FN) and +P=TP/(TP+FP). Here, TP is the number of true positives (*a* wave detected as an *a* wave), FN is the number of false negatives (*a* wave has not been detected), and FP is the number of false positives (non-*a* wave detected as an *a* wave). The SE reported the percentage of true *a* waves that were correctly detected by the algorithm. The +P reports the percentage of the detected *a* waves that were true *a* waves. Similarly, the same statistical measures were used for evaluating the *b* waves.

The function of the *a* wave detector (cf. pseudocode of Algorithm 1) has five inputs: the PPG signal (PPG_signal_), frequency band (*F*_1_– *F*_2_), event-related durations *W*_1_,*W*_2_, and the offset (*β*). Any change in these parameters will affect the overall performance of the proposed algorithm. These parameters are interrelated and cannot be optimized in isolation. A rigorous optimization via brute-force search, over all parameters, was conducted (cf. Table 1). This is a time-consuming process, but it is required before making definitive claims. The data used in this training phase were the PPG signals measured at after 1 hour of exercise. Optimization of the beat detector’s spectral window for the lower frequency resulted in a value within 0.5–1 Hz with the higher frequency within 7–15 Hz. The window size of the first moving average (*W*_1_) varied from 100 ms to 200 ms, whereas the window size of the second moving average (*W*_2_) varied from 1000 ms to 1.250 s. The offset *α* was tested over the range 0–10% of the mean value of the squared filtered PPG signal. The QRS complex corresponds roughly to the systolic duration (*a* wave duration) in APG, which is 100±20 ms in healthy adults [25]. Interestingly, the algorithm uses an optimal value of *W*_1_ (175 ms) corresponded to the *a* wave duration, and an optimal value of *W*_2_ (1000 ms) for the heartbeat duration. It is clear from Table 1 that the optimal frequency range for the systolic detection algorithm over the database was 0.5–15 Hz. Moreover, the optimal values for the moving-average window sizes and offset are *W*_1_=175 ms, *W*_2_=1000 ms, and *α*=0. The systolic algorithm was adjusted with these optimal parameters. Then, the detector was tested on two PPG datasets (PPG measured at rest and after 2 hours of exercise) without any further adjustment.

**Table 1:** **A rigorous optimization over all parameters of the** ***a*** **wave detection algorithm: frequency band,** ***W***_**1**_ **,** ***W***_**2**_ **, and the offset** ***β***

Iterations	Frequency band	***W***_1_	***W***_2_	Offset (%)	SE (%)	+P (%)	Overall accuracy (%)
1	0.5–15 Hz	35	200	0	99.72	100.00	99.86
2	0.5–11 Hz	25	200	0	99.92	99.78	99.85
3	1–15 Hz	35	200	0	99.68	100.00	99.84
4	0.5–13 Hz	35	200	0	99.67	100.00	99.84
5	0.5–14 Hz	20	220	0	100.00	99.64	99.82
6	1–14 Hz	35	200	0	99.64	100.00	99.82
7	0.5–14 Hz	20	200	0	99.92	99.71	99.82
8	0.5–14 Hz	20	210	0	99.92	99.71	99.82
9	0.5–13 Hz	25	200	0	99.84	99.78	99.81
10	1–14 Hz	25	200	0	99.84	99.78	99.81
11	1–14 Hz	25	210	0	99.84	99.78	99.81
12	0.5–13 Hz	20	200	0	99.92	99.66	99.79
13	1–15 Hz	30	200	0	99.75	99.82	99.79
14	0.5–15 Hz	30	200	0	99.68	99.89	99.78
15	1–9 Hz	35	200	0	99.55	100.00	99.78
16	0.5–12 Hz	25	220	0	100.00	99.55	99.78
17	0.5–14 Hz	35	200	0	99.54	100.00	99.77
18	1–15 Hz	30	250	0	99.75	99.79	99.77
19	0.5–15 Hz	25	200	0	99.92	99.61	99.76
20	0.5–12 Hz	25	200	0	99.84	99.68	99.76
.	.	.	.	.	.	.	
.	.	.	.	.	.	.	
.	.	.	.	.	.	.	
5606	0.5–8 Hz	40	230	10	90.22	99.88	95.05
5607	0.5–7 Hz	40	230	9	89.80	99.88	94.84
5608	0.5–7 Hz	40	240	9	89.96	99.68	94.82
5609	0.5–7 Hz	40	230	10	89.21	99.88	94.55
5610	0.5–7 Hz	40	240	10	89.38	99.68	94.53

All possible combinations of parameters (5,610 iterations) have been investigated and sorted in descending order according to their overall accuracy. The data used in this training phase were PPG measured after 1 hour of exercise, with 885 heartbeats. The overall accuracy is the average value of SE and +P.

## Results and discussion

Based on the parameter optimization step, the value of *α*=0, which means there is no need for an offset to improve the detection rate, as it was required in detecting QRS in ECG signals [21] and systolic peaks in PPG signals [20]. This is perhaps because of the sharp clear peak (high amplitude) of the *a* wave compared to the other APG waves (*c*, *d*, and *e* waves).

**Figure 7:**
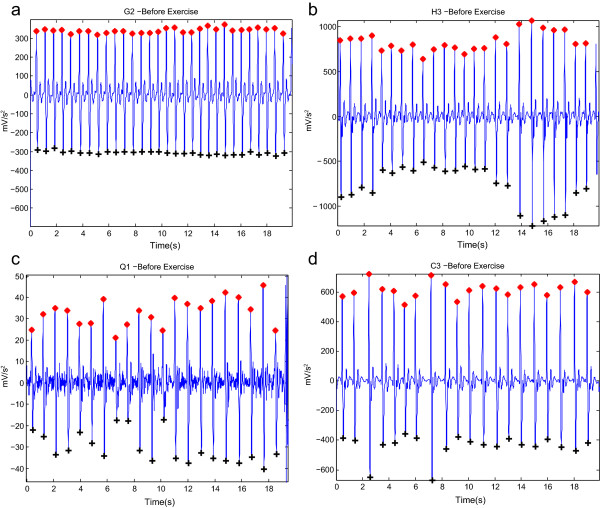
**Detected** ***a*** **and** ***b*** **waves in APG signals at rest (before exercise).** It contains **(a)** stationary signals, **(b)** non-stationary signals, **(c)** low amplitudes, and **(d)** irregular heart rhythm. Here, ‘red asterisk’ represents the detected *a* wave and ‘black plus sign’ represents the detected *b* wave by the proposed algorithm.

**Figure 8:**
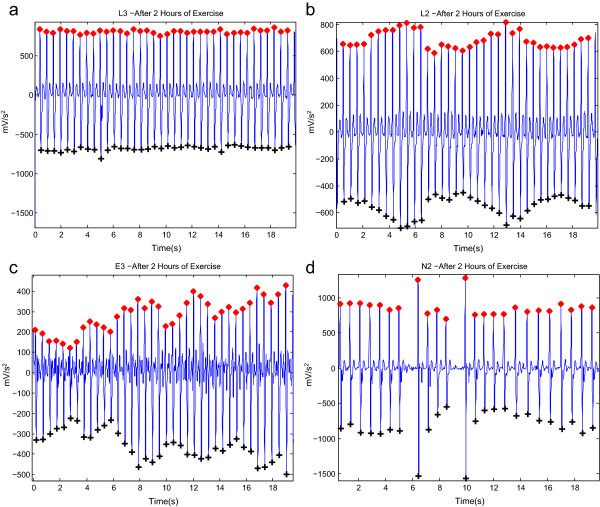
**Detected** ***a*** **and** ***b*** **waves in APG signals after 2 hours of exercise.** It contains **(a)** stationary signals, **(b)** non-stationary signals, **(c)** low amplitudes, and **(d)** irregular heart rhythm. Here, ‘red asterisk’ represents the detected *a* wave and ‘black plus sign’ represents the detected *b* wave by the proposed algorithm.

The *a* wave detection algorithms were tested on 27 subjects, with the APG signals measured before exercise and after 2 hours of exercise; with a total of 54 recordings, as shown in Table 2. The main objective was to evaluate the robustness of the algorithms against the non-stationary effects, low SNR, and high heart rate exhibited after exercise in conditions of heat stress. Under normal conditions, analyzing stationary APG signals is straightforward; as *a* waves have similar amplitudes, the statistical characteristics of the signals (i.e., mean and standard deviation) do not change appreciably with time, and a simple threshold level can effectively detect *a* waves. Figures 7(a) and 8(a) represent the APG signals with stationarity effects for volunteer G2 (before exercise) and L3 (after 2 hours of exercise)—all *a* waves are almost straight-lined. By contrast, under stress, APG signals become non-stationary, which makes analysis difficult since the standard deviation changes with time—note that *a* wave amplitudes vary with time and simple level thresholds cannot optimally detect *a* waves. This has a negative effect on detection algorithm performance, which is clearly seen in Table 3 when the nine amplitude-dependent algorithms were applied to the APG signals. Moreover, Matsuyama [5] reported that none of the nine amplitude-dependent algorithms achieved acceptable *a* wave detection rates even after optimizing the threshold values. Most of these nine algorithms, such as AF2, AF3, FD1, FD2, DF1, and FS1, strictly followed the morphology of the QRS complex. However, it is clear that amplitude-dependent algorithms are not optimal methodologies for detecting *a* waves in APG signals under varying conditions.

**Table 2:** **Performance of the proposed** ***a*** **wave detection algorithm on the testing dataset (APG signals measured at rest and after 2 hours of exercise)**

	Before exercise						After 2 hours of exercise					
Record	No of beats	TP	FP	FN	SE (%)	+P(%)	No of beats	TP	FP	FN	SE (%)	+P(%)
**A1**	26	26	0	0	100	100	43	41	0	2	95.34	100
**A2**	24	24	0	0	100	100	47	47	0	0	100	100
**B1**	17	17	0	0	100	100	44	43	0	1	97.72	100
**B2**	26	26	0	0	100	100	38	38	0	0	100	100
**C2**	20	20	0	0	100	100	37	37	0	0	100	100
**C3**	20	20	0	0	100	100	23	23	0	0	100	100
**D2**	22	22	0	0	100	100	39	39	0	0	100	100
**D3**	19	19	0	0	100	100	27	27	0	0	100	100
**E1**	22	22	0	0	100	100	30	30	0	0	100	100
**E2**	22	22	0	0	100	100	30	30	0	0	100	100
**E3**	19	19	0	0	100	100	38	38	0	0	100	100
**G2**	30	30	0	0	100	100	49	48	0	1	97.95	100
**G3**	19	19	0	0	100	100	42	41	0	1	97.61	100
**H3**	23	23	0	0	100	100	32	32	0	0	100	100
**I1**	22	22	0	0	100	100	35	35	0	0	100	100
**I2**	17	17	0	0	100	100	31	31	0	0	100	100
**J2**	23	23	0	0	100	100	41	41	0	0	100	100
**L2**	24	24	0	0	100	100	37	37	0	0	100	100
**L3**	24	24	0	0	100	100	39	39	0	0	100	100
**N2**	18	18	0	0	100	100	24	24	0	0	100	100
**N3**	20	20	0	0	100	100	31	31	0	0	100	100
**O1**	24	24	0	0	100	100	33	33	0	0	100	100
**O2**	17	17	0	0	100	100	34	34	0	0	100	100
**P1**	26	26	0	0	100	100	34	34	0	0	100	100
**P2**	20	20	0	0	100	100	34	34	0	0	100	100
**Q1**	22	22	0	0	100	100	28	28	0	0	100	100
**Q2**	18	18	0	0	100	100	36	36	0	0	100	100
27 volunteers	584	584	0	0	100	100	956	951	0	5	99.57	100

The PPG signals were collected from 27 subjects for 20 seconds during the 5 minutes break between each exercise [5]. To compare the performance of the proposed algorithm with the nine algorithms [5], two statistical measures were used: SE=TP/(TP+FN) and +P=TP/(TP+FP), where TP is the number of true positives (*a* wave detected as *a* wave), FN is the number of false negatives (*a* wave has not been detected), and FP is the number of false positives (non-*a* wave detected as *a* wave).

**Table 3:** **Comparison of different** ***a*** **wave detection performance on the testing dataset (APG signals measured at rest and after 2 hours of exercise)**

Algorithm	TP (%)	FN (%)	FP (%)	SE (%)	+P (%)	Threshold values		
						THR _1_	THR _2_	THR _3_
**Proposed**	100	0.32	0	99.78	100	*M* *A*_beat_+*α*	*W*_2_	NA
**algorithm**								
**AF1**	69.5	7.5	30.5	90.25	69.5	0.31	0.0001	-0.001
**AF2**	0.018	0.27	99.98	6.25	0.018	0.21	0.75	NA
**AF3**	0	0	100	NaN	0	62	NA	NA
**FD1**	0.27	2.8	99.73	8.79	0.27	0.099	NA	NA
**FD2**	0	0	100	NaN	0	150	NA	NA
**DF1**	0	0	100	NaN	0	21	NA	NA
**DF2**	48.8	14.2	51.2	77.46	48.8	1	0.06	NA
**FS1**	2.42	0.3	97.58	88.97	2.42	154.5	NA	NA
**FS2**	42.46	6.9	57.54	86.02	42.46	0.55	0.47	NA

The PPG signals were collected from 27 subjects for 20 seconds during the 5 minutes break between each exercise [5]. To compare the performance of the proposed algorithm with the nine algorithms [5], two statistical measures were used: SE=TP/(TP+FN) and +P=TP/(TP+FP), where TP is the number of true positives (*a* wave detected as *a* wave), FN is the number of false negatives (*a* wave has not been detected), and FP is the number of false positives (non-*a* wave detected as *a* wave). Here, NA stands for Not Applied, while NaN stands for Not-a-Number.

**Figure 9:**
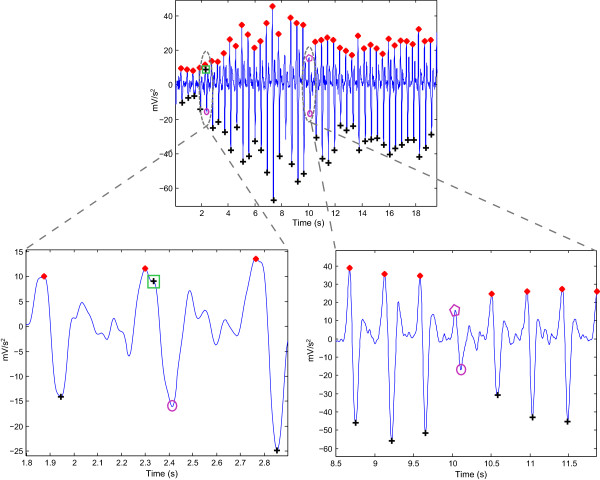
**Instances of failure occurring with the proposed algorithm (subject: A1 after 2 hours of exercise).** Here, ‘red asterisk’ represents the detected *a* wave and ‘black plus sign’ represents the detected *b* wave by the proposed algorithm. The purple pentagon represents a false negative for the *a* wave, while purple circle represents the false negative for the *b* wave. The green square represents the false positive of the *b* wave, which was the only false positive inccurred by the proposed *b* detection algorithm in the testing dataset.

The proposed algorithm scored the highest sensitivity and positive predictivity rates when compared to the nine algorithms. The proposed algorithm appears to be more robust against effects of post-exercise measurement non-stationarity. The results show that the proposed method was able to detect *a* waves correctly in non-stationary APG signals before exercise, as shown in Figure 7(b), and after 2 hours of exercise, as in Figure 8(b). Moreover, the proposed algorithm was also able to detect *a* waves correctly in low amplitude APG signals (small voltage), as shown in Figure 7(c), and after 2 hours of exercise, as in Figure 8(c). However, the algorithm did incur a few instances of failure, with exactly five FNs, as shown in Table 3. The cause of the failure was due to the sudden drop in amplitude of the *a* waves in heat-stressed APG signals (cf. Figure 9). The proposed method, however, handled varying amplitudes well compared to the other nine algorithms. In fact, it is clear that the proposed algorithm is more amplitude-independent and was able to detect the *a* waves in various voltage ranges.

The analysis of a regular heart rhythm is simple, as the *a* waves are repeated with an equally spaced pattern. This regularity helps the time-domain threshold methodologies to detect *a* waves successfully. The regular heart rhythm is called the normal sinus rhythm in APG signals [24], which means the rhythm is constant and the occurrence of the next beat is predictable. The proposed algorithm easily detects *a* waves correctly in APG signals with a regular heart rhythm, as shown in Figure 7(a,b,c). The sensation of an irregular heart rhythm is usually related to either premature beats or atrial fibrillation. The proposed algorithm also successfully detected the *a* waves with premature beats in both conditions at rest and after exercise, as shown in Figures 7(d) and 8(d).

As the detection of *b* waves depends on the detection of *a* waves, the performance of the *b* wave detection scored almost the same result as the *a* detection algorithm. Because the proposed *b* detection incurred only one instance of failure, which is a TP shown in Figure 9, the +P becomes 99.95%. This result reflects the robustness of the proposed *b* detection algorithm against noisy APG signals.

## Limitations of the study and future work

One of the next steps regarding the results of this study is to examine the correlation of the *a*/*b* ratio—based on the accurately detected *a* and *b* waves—using APG signals with age, body mass index, and core temperature. Moreover, there is a need for developing an algorithm that detects the *c*, *d*, and *e* waves.

The proposed algorithm was only tested on normotensive young subjects. The physiology of the photoplethysmogram significantly changes according to health status. As such, the robustness of the proposed algorithm needs to be verified by a study in unhealthy subjects—to diagnose and monitor abnormalities such as arrhythmia, hypertension, diabetes and hyperlipidemia.

It is important to note that the number of PPG records (total of 27) used in the training was modest. A larger sample size and a more diverse data set are needed in order to generalize the findings of this study. Moreover, sampling the PPG signals at a higher rate (above 200 Hz) is required to capture the *b* waves with greater fidelity. The evaluation of *a* wave detection was challenging in this study because the number of annotated beats did not allow all possible morphologies found in APG signals under conditions of heat stress to be well represented. To our knowledge, there is no available APG database measured after heat stress that would allow a more thorough assessment and comparison of the tested algorithms.

## Conclusion

For all nine QRS algorithms, the detection errors arose from a variety of factors including the existence of irregular heartbeats, low-amplitude peaks, and signals with non-stationary effects. The application of an event-related dual moving average would allow the accurate, computationally simple algorithm we propose to be used for real-time applications and the processing of large databases. A detection algorithm for *a* waves in APG signals measured after exercise has not been previously addressed in the literature, with the exception of the work of Matsuyama. However, it has been demonstrated that a robust algorithm can be developed for detecting *a* waves in APG signals collected in a noisy environment with high-frequency noise, low amplitude, non-stationary effects, irregular heartbeats, and high heart rates. The *a* wave detection algorithm was evaluated using 27 records, containing 1,540 heartbeats (584 heartbeats measured at rest and 956 heartbeats measured after 2 hours of exercise), with an overall sensitivity of 99.78%, and the positive predictivity was 100%, while the *b* detection algorithm scored an overall sensitivity of 99.78% and a positive predictivity of 99.95%.
